# The Role of the Critical Coronoid Angle in Simple Elbow Dislocation: A Computed Tomography-Based Index to Stratify Elbow Dislocation Risk

**DOI:** 10.3390/jcm14103323

**Published:** 2025-05-09

**Authors:** Paolo Arrigoni, Francesco Luceri, Enrico Rosagrata, Salvatore Sorrentino, Dario Polli, Andrea Zagarella, Simone Cassin, Valeria Vismara, Alessandra Colozza, Carlo Zaolino, Pietro Simone Randelli

**Affiliations:** 1 Clinica Ortopedica, Azienda Socio Sanitaria Territoriale Centro Specialistico Ortopedico Traumatologico Gaetano Pini-CTO, Piazza Cardinal Ferrari 1, 20122 Milan, Italy; f.luceri88@gmail.com (F.L.);; 2 Scuola di Specializzazione in Ortopedia e Traumatologia, Università degli Studi di Milano, Via Festa del Perdono 7, 20122 Milan, Italy; 3 Department of Physics, Politecnico di Milano, Piazza L. da Vinci 32, 20133 Milan, Italy; salvatore.sorrentino@polimi.it (S.S.);; 4 Consiglio Nazionale delle Ricerche—Institute for Photonics and Nanotechnologies (CNR-IFN), Piazza L. da Vinci 32, 20133 Milan, Italy; 5 U.O.C. Radiodiagnostica, Azienda Socio Sanitaria Territoriale Centro Specialistico Ortopedico Traumatologico Gaetano Pini-CTO, Piazza Cardinal Ferrari 1, 20122 Milan, Italy; 6 Unità Operativa Ortopedia e Traumatologia, Ospedale Civile di Faenza, Viale Stradone, 9, 48018 Faenza, Italy; 7 Laboratory of Applied Biomechanics, Department of Biomedical Sciences for Health, Università degli Studi di Milano, Via Mangiagalli 31, 20133 Milan, Italy; 8 Research Center for Adult and Pediatric Rheumatic Diseases (RECAP-RD), Department of Biomedical Sciences for Health, Università degli Studi di Milano, Via Mangiagalli 31, 20133 Milan, Italy; 9 U.O.C. 1° Clinica Ortopedica, ASST Centro Specialistico Ortopedico Traumatologico Gaetano Pini-CTO, Piazza Cardinal Ferrari 1, 20122 Milan, Italy

**Keywords:** elbow dislocations, CT scans, coronoid angle, instability, indexes

## Abstract

**Introduction:** Elbow primary stability is guaranteed by the anatomical congruency between the humeral trochlea and the greater sigmoid notch (GSN). Elbow dislocation typically occurs in a semi-extended position, but computed tomography (CT) scans are typically acquired at 90° of elbow flexion, which may misleadingly suggest that the apex of the coronoid aligns with the trochlear center of rotation. This study aims to evaluate the anatomical features of the coronoid and GSN in a dislocated versus non-dislocated group, demonstrating that a more prominent coronoid process is more commonly observed in elbows without dislocation compared to those with dislocation. **Materials and Methods:** A total of 50 CT scans, equally divided between dislocated elbows and non-dislocated elbows, were analyzed, and the critical coronoid angle (CCA) was measured on a specific slice of the CT scan (level of evidence III). The CCA was calculated from two lines that arise in the center of the GSN, with the first one crossing the coronoid tip and the second parallel to the posterior olecranon cortex. **Results:** A significant difference in the CCA (*p* < 0.001) between the two groups was highlighted. In particular, it was found that 14/25 patients from the dislocated elbow group had a CCA below or equal to 27°, and all the non-dislocated subjects had a CCA ≥ 27°. These preliminary results suggest that a CCA ≤ 27° could be a threshold for requiring further imaging of soft tissues or closer follow-up. This may result from either a hypoplastic coronoid process or a decreased concavity of the GSN. Based on the CCA values, a logistic regression model (DAM model) was proposed to associate a coefficient of protection to the CCA, the angle of flexion during dislocation (FdD), and a parameter X, which is a factor that encompasses the contribution of soft tissues. **Conclusions:** A low CCA is statistically more frequent in dislocated elbows versus non-dislocated ones, creating a specific anatomical condition. The CCA should be carefully evaluated by elbow surgeons to guide patient-specific treatment. The DAM model can permit the stratification of patients eligible for further diagnostic analysis.

## 1. Introduction

Elbow primary stability is guaranteed by anatomical congruency between the humeral trochlea and the greater sigmoid notch (GSN) [1,2]. For this reason, the role of the olecranon and the coronoid is critical [1,3,4]. The role of the GSN on elbow stability has been extensively studied on cadavers, with significant results that are not easily transferrable in clinical practice [5,6].

Elbow dislocations can be classified as simple, when only soft tissues are damaged, or complex, when an associated fracture is present. A complex dislocation is characterized by one or more associated fractures, and often requires surgical intervention to restore joint stability and congruity [7,8]. The majority of simple dislocations are posterolateral, resulting from a valgus–supination–hyperextension mechanism typically following a fall onto an outstretched hand with the elbow in partial extension [9].

Classification systems also consider the direction of dislocation and the specific osseous and ligamentous structures involved, for example, the coronoid or radial head fractures in the case of complex dislocations [10,11].

Simple elbow dislocation often causes the sequential disruption of soft tissue stabilizers, with often high-grade or complete lesions of the lateral ulnar collateral ligament (LUCL) [12,13,14]. Injury to the LUCL typically occurs in the context of elbow dislocations or traumatic varus forces. It is often the first structure disrupted in the sequential lateral-to-medial failure described in posterolateral dislocations [15,16]. In case of simple elbow and small coronoid fracture dislocations, ligamentous injury is significant, and this could facilitate posterolateral capsular avulsion [17]. The impact of the radial head and coronoid process on the humerus frequently leads to their fracture [18,19]. These patterns require proper management that involves an accurate assessment, including neurovascular examination, radiographic evaluation, and potential CT scans, and often lead to surgical treatment.

CT scans are normally performed after elbow reduction at 90° of flexion, while elbow dislocations typically occur in a semi-extended position (30–45° of flexion) [4,20]. This may create the false idea that the apex of the coronoid provides an anterior buttress to the center of rotation of the distal humerus, whereas its barrier effect decreases dramatically in the semi-extended position

The purpose of this study is to evaluate the anatomical features of the coronoid and GSN, and compare dislocated versus non-dislocated elbows. We hypothesize that a more prominent coronoid would be more frequently found in non-dislocated elbows compared to dislocated ones.

## 2. Materials and Methods

A cohort of CTs performed between January 2019 and January 2021 was retrospectively selected from patients who sustained dislocation and, despite an initial suspected fracture, classified as simple dislocation (dislocated group). Meanwhile, a control group of patients without any history of instability or fractures was selected, defining a level III of evidence. Within the first group, only CT scans obtained after reduction from dislocation were enrolled. Patients with signs or a history of previous elbow fractures, degenerative or rheumatic pathologies, or previous surgery were excluded.

Each patient and CT scan was anonymized through an alphanumeric code. All the procedures were performed according to the Helsinki Declaration (1964) and its later amendment and to the ethical standards of the institutional and/or national research committee. This study received approval from our Institute Ethical Committee (Session 595_2019).

**Figure 1:**
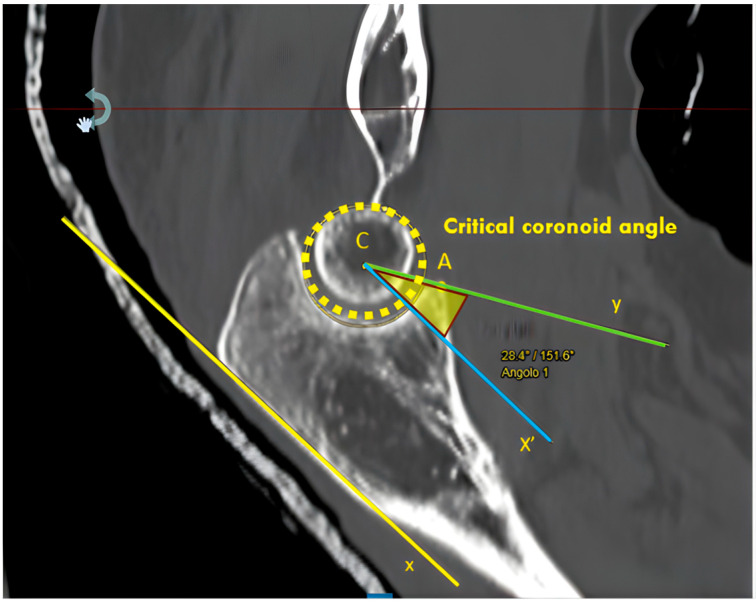
MPR projections using RadiAnt DICOM Viewer for coronoid angle calculation. The coronoid angle was determined by two lines arising from the GSN center (C dot): the first one (Y, green line) crosses to the coronoid tip (A dot), and the second one (X’, blue line) was drawn parallel to the posterior olecranon cortex (x, yellow line). MPR, multiplanar reconstruction; GSN, greater sigmoid notch.

A new CT index, named critical coronoid angle (CCA), was measured on a specific cut of the CT scan [6,21] using a multiplanar reconstruction tool (Radiant DICOM Viewer with its Multiplanar Reconstruction (MPR) function). The standard plane was defined using cross-sectional images of the GSN, lying on a plane defined by the tip of the olecranon and the tip of the coronoid and parallel to both the coronal plane and the GSN axis, which is tilted concerning the sagittal axis. The CCA was calculated as the angle between two lines arising from the GSN center (Figure 1); the first one intercepts the coronoid tip (A dot), and the second line is drawn parallel to the posterior olecranon cortex (Figure 1).

All the CT scans were evaluated independently by three expert elbow surgeons. The sequence of CT scans at presentation was randomized and blinded from details to all the examiners. Each observer repeated the assessment twice, with a 15-day interval between the assessments for bias reduction. The mean results of these measurements were reported and analyzed.

Statistical analyses were performed using IBM SPSS Statistics for Windows, Version 25.0 (Armonk, NY, USA: IBM Corp., released 2017). The Shapiro–Wilk test was used to evaluate the normality of data distributions [22]. Differences between groups were evaluated with an unpaired Student’s *t*-test or a Mann–Whitney test according to the characteristics of the data distribution for continuous variables (samples with normal or with non-normal distributions, respectively). The reproducibility was evaluated with the intraclass correlation coefficient (ICC) with a 95% confidence interval.

Moreover, the logistic regression analysis was performed in Python 3.9 using the SciPy library version 1.12.3. Based on this analysis, we proposed and modeled a statistical protection coefficient for dislocation, the DAM ranging from −1 to 1, which can be used as a coefficient indicating the risk of dislocation associated with a given CCA, as discussed in the Results section. Therefore, a positive DAM indicates a high risk of dislocation associated with the corresponding CCA, while a negative DAM indicates a lower risk. Moreover, the larger the DAM, the higher its associated dislocation risk. The predictive power of the DAM coefficient was evaluated using leave-one-out cross-validation (LOOCV) [23]. LOOCV is a widely recognized validation method, particularly effective for small datasets, where maximizing data utilization is critical [23].

In LOOCV, the model is trained iteratively by leaving out one observation at a time for validation while using the remaining n−1 samples for model fitting. This process is repeated for each data point in the dataset, ensuring that every observation is tested on unseen data. The overall performance is then computed as the average across all iterations, providing a nearly unbiased estimate of the model’s predictive ability.

In this study, LOOCV was applied to assess the predictive accuracy of the developed model. Specifically, each subject was sequentially excluded from the fitting set and used for validation. This process was repeated until every subject in the dataset was validated.

Given the relatively small size of the dataset, LOOCV was particularly well-suited for this analysis, as it ensured that the model was trained and tested on nearly all available data, reducing the risk of overfitting and enhancing generalizability [23].

Based on a preliminary analysis performed on the first six measurements per group and considering a desired power of 80% and a significance level of 5% (two-sided comparison of proportions), a sample size of 50 patients (25 in each group) was considered sufficient to test a difference of at least 10% in CCA values between the groups.

## 3. Results

A total of 50 elbow CT scans, 25 from dislocated patients and 25 from controls, were analyzed. Out of the 25 CTs from the non-dislocated group, 11 (44%) were of the left elbow versus 12 (48%) from the dislocated group. In both cohorts, 14 were women (56%) and 11 (44%) were men. A mean age of 46 years was found for the non-dislocated group (range 22–64 years) and 50 for the dislocated group (range 18–81 years). No significant differences were highlighted between the study groups (*p* > 0.05).

According to the Shapiro–Wilk test, the CCA was normally distributed in both groups.

A total of 14 out of 25 patients in the dislocated group presented with a CCA ≤ 27° (56%), and 10 out of 25 patients had a CCA between 27° and 38° (40%), with only one patient with a CCA ≥ 38°. Conversely, all the non-dislocated patients showed a CCA > 27° (100%), and 11 out of 25 presented with a CCA ≥ 38° (44%).

Interpolation curves were then plotted to two Gaussians, with apexes at 27° of CCA for the dislocated group and 38° of CCA for the non-dislocated one. The lower CCA value in the former group indicates a less restrictive GSN; conversely, the higher CCA value in the latter group suggests a more restrictive GSN.

A receiver operating characteristic (ROC) analysis was performed, and the area under the curve (AUC) was calculated as 0.940. This value suggests that the CCA could be considered a reliable test with a good to excellent ability to discriminate between individuals whose elbows have a more or less prominent coronoid. A Youden index of 0.72, corresponding to a CCA of 30.5°, was also calculated.

Based on the CCA values, we proposed a variant of a logistic regression model [23] to associate a coefficient of protection (DAM) to the CCA, the angle of flexion during dislocation (FdD), and a parameter X, which is a factor that encompasses the contribution of soft tissues.

The regression model to fit can be written as

(1) $$DAM=\frac{2}{1+e^{-(\beta_{0}+\beta_{1}*CCA+\beta_{2}*FdD+\beta_{3}*X)}}-1$$

where *β*_0_ is the intercept coefficient of the regression, *β*_1_, *β*_2_, and *β*_3_ are respectively the regression coefficients for the variables CCA, FdD, and X, and DAM is a risk coefficient ranging between −1 and 1.

We fitted the proposed regression model to our dataset, which contains the CCAs of a cohort of 25 patients for the dislocation group and 25 patients for the non-dislocation group; for fitting purposes, we assigned DAM = −1 for patients in the dislocation group and DAM = 1 for patients in the non-dislocation group. FdD and X were ignored because they were not collected in our cohort; thus, only the CCA variable was considered in the logistic regression analysis.

In this case, the logistic regression model was simplified to

(2) $$DAM=\frac{2}{1+e^{-(\beta_{0}+\beta_{1}*CCA)}}-1$$

Performing a least square minimization of the fitting equation to evaluate *β*_0_ and *β*_1_, we obtained the following coefficient values: *β*_0_ = −12.933 and *β*_1_ = 0.405. For every patient having a given CCA, using the reduced model, we can calculate the DAM coefficient.

Based on the regression model proposed, it is possible to set a threshold for the DAM index value to address different clinical choices based on the DAM value above or below the threshold.

**Figure 2:**
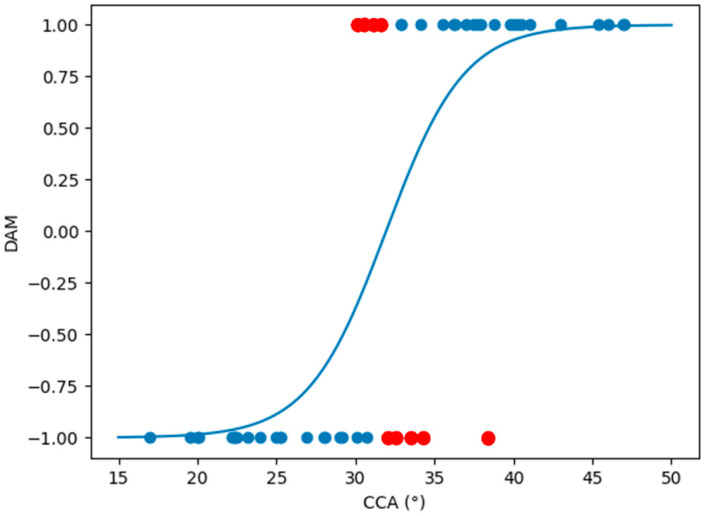
Best fit logistic regression curve of our cohort data using CCA as only regressor. Blue dots are data points correctly classified by the logistic regression model, while red dots are misclassified data points. CCA, critical coronoid angle.

To assess the predicting efficiency of this reduced regression and to corroborate its use, we performed a LOOCV on this model [24]. Leave-one-out cross-validation, as described in the Materials and Methods section, is a model evaluation technique where each data point in the dataset is systematically held out while the model is trained on the remaining data points, allowing for an evaluation of the model performance across all individual instances in the dataset. For this LOOCV, we assumed that a given patient was part of the dislocated group if the DAM index was <0 and part of the non-dislocated group if the DAM index was >0, and then we compared this prediction with the true patient group. Using this procedure and employing only CCA as a regressor, the predictive model accuracy was 82%, thus allowing us to conclude that the CCA factor alone is sufficient to achieve a high performance in terms of patient dislocation classification. Figure 2 shows the logistic regression curve that best fits the CCA data in our cohort; the blue dots are the data points that were correctly classified by the regression, and the red dots are the data points that were misclassified after a comparison with the true patient group.

**Figure 3:**
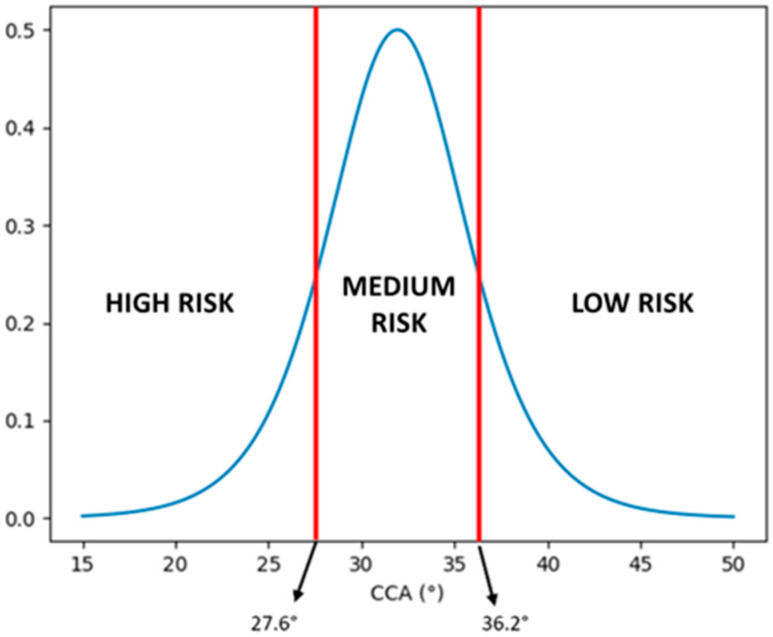
Derivative of the reduced logistic regression function used to model the DAM factor (blue plot). The two red lines indicate the two values, at 27.6° and 36.2° for the CCA angle, in which the value of the derivative is equal to half of the maximum of the derivative itself. These two limit values define the high risk of dislocation band, the medium risk of dislocation band, and the low risk of dislocation band. CCA, critical coronoid angle.

Using the derivative of the reduced logistic regression function used to model the DAM factor, it is possible to determine three bands in the DAM value to distinguish between a high risk of dislocation, a medium risk of dislocation, and a low risk of dislocation. The three bands were chosen using the full width at half maximum (FWHM) of the derivative function, as indicated in Figure 3, which shows the derivative of the logistic function used and the thresholds that define the bands.

In particular, the maximum of the derivative is at 0.5, and the two CCA angles at which the derivative is equal to the half maximum value (0.25) are at 27.6° and 36.2°, which correspond respectively to DAM values of –0.704 and 0.704. We can define a high risk of dislocation for DAM values between –1 and –0.704, a medium risk between –0.704 and 0.704, and a low risk between 0.704 and 1.

The identification of the three DAM risk regions—high, medium, and low—is intended for descriptive purposes and does not contribute to the predictive capacity of the current logistic regression model. The model’s predictive performance is exclusively evaluated using the DAM coefficient within a binary classification framework, as the available dataset only provides binary outcome labels. The identified thresholds based on the derivative function are presented as an exploratory tool to conceptually illustrate how DAM values might correspond to varying degrees of dislocation risk. However, these bands are not utilized in the model’s predictive analysis and are not validated for risk stratification. Future studies with more granular data could potentially expand upon this framework to support multi-class risk classification.

## 4. Discussion

The main finding of this study is a significant difference in the CCA between the dislocated and the non-dislocated elbows.

Some indexes, based on the elbow’s anatomical features, have already been described [25,26,27,28]. However, none of them give clear therapeutic guidance. Currently, there are no objective radiological findings that are able to predict the intrinsic level of elbow instability. This study stratifies the anatomical difference between dislocated elbows and non-dislocated ones, as proposed for other anatomical regions [27,29]. Whilst bone congruency is only one aspect of elbow joint stability, a higher joint congruency is very likely associated with a lower dislocation risk [30]. Ligamentous status cannot be assessed through CT; however, it can be assumed that dislocated elbows suffer from ligamentous ruptures, as previously demonstrated [31,32]. We decided to evaluate only bony-dependent radiological parameters, independent from ligamentous structures, to avoid soft tissue biases. Tools such as dynamic ultrasound, fluoroscopy examination, and magnetic resonance imaging (MRI) could be very helpful in further studies to directly assess elbow stability and soft tissue damage [33,34,35,36].

While posterolateral rotatory elbow dislocation occurs in a semi-extended position (flexion of 30–45°), post-reduction elbow CT scans are usually performed at 90° of flexion [4,20,37]. This practice leads to a very difficult assessment of the effective GSN restriction without dedicated radiological indexes, such as the CCA. At 90° of flexion, the apex of the coronoid in a lateral view is very likely to be superior to the center of rotation of the distal humerus, giving a false sense of joint congruency. However, the CCA can help us depict a spectrum of stability: patients with a less prominent coronoid could be more prone to dislocation or at least subluxation, while those with a more prominent coronoid could be more protected. Elbow extension progressively decreases the coverage provided by the coronoid, which also is under stress by sustaining increasing forces against posterior dislocation [38]. Furthermore, axial loads exert more force on the coronoid with increasing extension from the base to the apex [38].

The role of the cartilaginous cap at the level of the apex of the coronoid must be further investigated. A few millimeters difference in the height of the coronoid can lead to dramatically different CCA values and could mean the difference between a stable elbow and one more at risk for dislocation. Potentially, further studies focusing on the elbow’s position at the moment of trauma could let clinicians estimate the dimension of the cartilaginous cap at the apex of the coronoid, verifying its impact [39].

**Figure 4:**
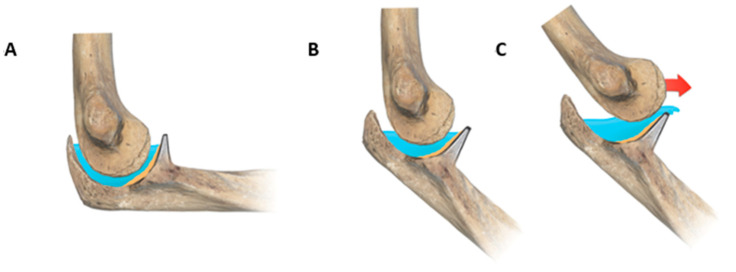
Graphic representation of the DAM index elbow theory. At 90° flexion, the tip of the coronoid is likely superior to the trochlea rotation center (**A**). If the elbow is semi-flexed, the center of the trochlea is out of the water (**B**); therefore, even a smaller force could cause elbow dislocation (**C**).

A model of the CCA can be imagined as a mountain basin containing a body of water that is reoriented because of gravity and becomes completely horizontal independently from the mountain profile inclination (Figure 4). Within this model, the apex of the coronoid could be considered as the dam that prevents water extravasation, while the humeral trochlea could be thought of as a floating body. The higher the angle of the FdD or CCA, the greater the dam contributes to containing the water and vice versa.

The statistical model proposed above can be fully applicable once a study of a cohort of dislocated and non-dislocated patients determines the FdD value and the soft tissue contribution of X using the predictors CCA, FdD, and X. In this way, it would be possible to establish the relative importance of the predictors CCA, FdD, and X on the DAM risk of dislocation [40].

The energy of trauma acts like a landslide or an earthquake on the dam, depending on the intensity, and generates a wave disturbing the water. Severe trauma will invariably cause dislocation; however, for a limited trauma, a positive DAM can contribute to elbow stability, in which all the water remains; a subluxated elbow results in partial water loss; or a complete dislocation leads to severe water loss and rupture of the dam (Figure 4). As a consequence of the logistic regression model, the CCA could be interpreted as a predictive factor to stratify the risk of recurrent elbow dislocation, helping clinicians with diagnostic and therapeutic choices. A low CCA, for example, may suggest a more cautious rehabilitation protocol after the first episode of dislocation, extended soft tissue imaging, and closer follow-up appointments.

Several other parameters play an important role in elbow instability: age, sex, soft tissue injuries (e.g., ligaments and tendons), and, most of all, the trauma pattern that led to the elbow dislocation [14,41]. This study emphasized that bony anatomical parameters may also play a key role. Stratifying instability could aid the clinical practice of orthopedic surgeons for elbow instability. The model described above is particularly interesting following a first simple elbow dislocation in high-demand patients and may facilitate a better clinical, therapeutic, and preventive assessment.

This study is affected by several limitations: it is a retrospective study on a small series of patients, but the sample size was sufficient according to the power analysis performed. The CCA assessments did not take inter-observer variability into account, and the study exclusively utilized CT scans. Also, more comprehensive and prospective studies are necessary to confirm these results, evaluate the predictive role of the CCA and DAM index, and assess what the best preventive options are according to the patient’s bony anatomy.

## 5. Conclusions

The CCA is a new predictive tool that can help the physician depict a spectrum of elbow stability; a low CCA is statistically more frequent in dislocated elbows versus non-dislocated ones, creating a specific anatomical condition. Patients with a less prominent coronoid could be more prone to dislocation or at least subluxation, while those with a more prominent coronoid could be more protected. The CCA should be carefully evaluated by elbow surgeons to guide patient-specific treatment.

The DAM index proposed can offer an interesting and promising model to further stratify the patients eligible for further diagnostic analysis, but it will require further validation for its clinical application.

Further research on the DAM model and the CCA could lead to new insights into the stratification of elbow dislocation and stability.

## Data Availability

Data are available on request from the corresponding author.

## Abbreviations

Greater sigmoid notch (GSN), computed tomography (CT), critical coronoid angle (CCA), lateral ulnar collateral ligament (LUCL), multiplanar reconstruction (MPR), receiver operating characteristic (ROC), area under the curve (AUC), flexion during dislocation (FdD).
